# Does television viewing predict dietary intake five years later in high school students and young adults?

**DOI:** 10.1186/1479-5868-6-7

**Published:** 2009-01-30

**Authors:** Daheia J Barr-Anderson, Nicole I Larson, Melissa C Nelson, Dianne Neumark-Sztainer, Mary Story

**Affiliations:** 1 School of Kinesiology, University of Minnesota, 207 Cooke Hall, 1900 University Ave SE, Minneapolis, MN 55455, USA; 2 Division of Epidemiology and Community Health, School of Public Health, University of Minnesota, 1300 South 2nd Street, Suite #300, Minneapolis, MN 55454, USA

## Abstract

**Background:**

Prior research has found that television viewing is associated with poor diet quality, though little is known about its long-term impact on diet, particularly during adolescence. This study examined the associations between television viewing behavior with dietary intake five years later.

**Methods:**

Survey data, which included television viewing time and food frequency questionnaires, were analyzed for 564 middle school students (younger cohort) and 1366 high school students (older cohort) who had complete data available at Time 1 (1998–1999) and five years later at Time 2 (mean age at Time 2, 17.2 ± 0.6 and 20.5 ± 0.8 years, respectively). Regression models examined longitudinal associations between Time 1 television viewing behavior and Time 2 dietary intake adjusting for sociodemographic characteristics, Time 1 dietary intake, and Time 2 total daily energy intake.

**Results:**

Respondents were categorized as limited television users (*<*2 hours/daily), moderately high television viewers (2–5 hours/daily), and heavy television viewers (≥5 hours/daily). Among the younger cohort, Time 1 heavy television viewers reported lower fruit intake and higher sugar-sweetened beverage consumption than the other two groups. Among the older cohort, watching five or more hours of television per day at Time 1, predicted lower intakes of fruits, vegetables, whole grain and calcium-rich foods, and higher intakes of trans fat, fried foods, fast food menu items, snack products, and sugar-sweetened beverages (products commonly advertised on television) five years later.

**Conclusion:**

Television viewing in middle and high school predicted poorer dietary intake five years later. Adolescents are primary targets of advertising for fast food restaurants, snack foods, and sugar-sweetened beverages, which may influence their food choices. Television viewing, especially during high school, may have long-term effects on eating choices and contribute to poor eating habits in young adulthood.

## Background

Obesity and related health problems are increasing in adolescents in the United States. In the mid-1960s, less than 5% of 12–19 year olds were overweight [1]. Forty years later, national survey data indicate that 17% of adolescents are obese and another 17% are overweight [2]. On average, adolescents aged 14–18 years in the United States watch over 18 hours of television per week [3] and previous research has suggested that high rates of television viewing may be linked to obesity and adiposity [4]. During the time spent watching television, not only do adolescents expend little energy [5], but they are exposed to numerous advertisements that can influence the type and amount of food desired, requested, and consumed [4,6]. Fast foods, high-fat, high-sugar foods, and sugar-sweetened beverages are heavily advertised during prime time programs that target all age groups, including adolescents [7]. Exposure to food and beverage advertisements may foster unhealthy perceptions about food and nutrition [8], contribute to a significant proportion of total daily energy consumed while watching television [9], and promote weight gain [4].

The relationship between the sedentary behavior of television viewing and dietary behaviors has been explored in children and adolescents; however, most of this work has been cross-sectional in nature. Cross-sectional studies have found that television viewing behavior is associated with less than optimal eating behaviors, including lower fruit and vegetable intake [10,11], higher sugar-sweetened beverage consumption [11,12]; and higher fast food intake [11]. Little research has examined the longitudinal relationship between television viewing and diet. Two studies using data from Planet Health, a 19-month obesity reduction intervention for middle school students, found that each additional hour of television beyond the baseline number of hours watched resulted in a reduction of almost one fruit and vegetable serving per week [13] and increased servings of baked sweet goods, candy, fast food, fried potatoes, salty snacks, and sugar-sweetened beverages – foods commonly advertised on television [14]. We are unaware of any longitudinal studies that have examined the relationship between television viewing and diet in older adolescents.

The current study explores whether time spent watching television during middle school and high school is associated with eating behaviors five years later, as youth transition from middle school into high school and from high school to the post-high school years or young adulthood. The middle school and high school years are crucial developmental periods, as adolescents may form behaviors that continue throughout their adult lives. We hypothesized that adolescents who watched more television when they were younger would report poorer eating behaviors five years later.

## Methods

### Study design and population

Project EAT (Eating Among Teens) is a longitudinal study of socioenvironmental, personal, and behavioral determinants of dietary intake and weight status in adolescents. In Project EAT-I, middle and high school students (n = 4746) completed in-class surveys and anthropometric measures during the 1998–1999 academic year (Time 1). Five years later (2003–2004; Time 2), Project EAT-II re-surveyed respondents by mail as middle school students progressed from early adolescence to middle adolescence and entered high school (younger cohort), and the high school students progressed from middle adolescence to late adolescence and entered young adulthood (older cohort). Of the original Project EAT-I sample, 2516 participants completed surveys at Time 2; 32.0% (n = 805) were in the younger cohort (middle school students at Time 1 and high school students at Time 2) and 68.0% (n = 1711) were in the older cohort (high school students at Time 1 and young adults at Time 2). Only respondents who reported on their television viewing and dietary behaviors at both Time 1 and Time 2 were retained for the current analysis. The final samples were ethnically and socioeconomically diverse groups of 564 middle adolescents (younger cohort) and 1366 young adults (older cohort) for a total of 1930 participants (Table 1). The mean age of the younger cohort was 12.8 ± .7 years at Time 1 and 17.2 ± .6 years at Time 2. The mean age of the older cohort was 15.9 ± .8 years at Time 1 and 20.5 ± .8 years at Time 2. Study protocols for both waves of Project EAT were approved by the University of Minnesota's Institutional Review Board Human Subjects Committee.

**Table 1:** Sociodemographic characteristics of Project EAT sample at Time 2^1^

	**Younger Cohort** **n = 564**		**Older Cohort** **n = 1366**	
		
	n	%	n	%
**Gender**				
Male	249	44	608	45
Female	315	56	758	55
**Race/ethnicity**				
White	227	40	838	61
Black	120	21	180	13
Asian	122	22	236	17
Hispanic	43	8	58	7
Other^2^	52	9	54	4
**Socioeconomic status**				
Low	92	16	219	16
Low middle	105	19	263	19
Middle	179	32	336	25
Upper middle	102	18	361	26
High	86	15	186	14

^1 ^Data are unadjusted and weighted and represent only the participants included in this current study.

^2 ^Other race/ethnicity category includes Native American and mixed race participants.

### Measures

#### Time spent watching television at time 1

Items adapted from the Planet Health study were used to assess usual time spent watching television and videos [15]. Respondents were asked, "In your free time on an average weekday (Monday-Friday), how many hours do you spend watching TV & videos?" Response categories were 0, 1/2, 1, 2, 3, 4, and 5+ hours. A similar question was asked regarding weekend (Saturday or Sunday) television and video viewing habits. In Project EAT-I, two-week test-retest correlations for these survey items ranged from .66 to .80 [16]. The validation of similar items has been described in detail elsewhere [17]. Categorical responses were summed, weighted, and averaged according to weekday and weekend to represent the average number of hours per day spent watching television and videos. To provide a valid comparison with national guidelines on television viewing, respondents were categorized as limited television viewers (<2 hours/daily), moderately high television viewers (2–5 hours/daily), or heavy television viewers (≥5 hours daily). These cut points were based on American Academy of Pediatrics guidelines for daily television [18] and Kaiser Family Foundation's definition of heavy television use [3]. Throughout the manuscript, this variable is referred to as television viewing, although it also includes video watching.

#### Dietary intake and fast food intake at time 2

Dietary intakes (servings/day) of fruits, vegetables (excluding french fries), whole grains (excluding chips), snack foods, fried foods, sugar-sweetened beverages, and calcium-rich foods in the past year were measured using the Youth/Adolescent Food Frequency Questionnaire (YAQ). The reliability and validity of the YAQ have been examined in prior studies that concluded the tool provides acceptable estimates of dietary intake for adolescents [19-21]. Total calories and the percentages of total energy intake from total fat, saturated fat, and trans fat were also assessed using the YAQ. Past week frequency of eating from a fast food restaurant was assessed on the Project EAT survey. Respondents were asked, "In the past week, how often did you eat something from a fast food restaurant (like McDonald's, Burger King, Hardee's, etc)?" This assessment of fast food intake has not been rigorously validated, but is comparable to the measures used by several other studies to assess fast food intake in adolescents and adults [22-24]. Response options (never, 1–2, 3–4, 5–6, 7, or more than 7 times/week) were recoded (0, 1.5, 3.5, 5.5, 7, or 9 times/week) to create a score that represented the number of times eaten from a fast food restaurant in the past week.

#### Anthropometric variables

At Time 2, height (m) and weight (kg) were self-reported by survey respondents, which are highly correlated with objectively measured values [25]. Body mass index (BMI) was calculated as kg/m^2^.

#### Demographic variables

Respondents self-reported their age, gender, and race/ethnicity. Socioeconomic status (SES) was assessed by an algorithm based primarily on highest level of parental educational attainment, and also accounted for family eligibility for public assistance, eligibility for free or reduced-cost school meals, and parental employment status [26]. Data from Time 1 were used to define the demographic variables.

### Statistical analysis

All analyses were run separately for the younger and older cohorts. Multivariate regression models adjusted for gender, race/ethnicity, SES, Time 1 dietary intake, and Time 2 total daily energy intake were used to examine the association of Time 1 television viewing with Time 2 dietary intake outcomes. P-values from tests for linear trend across the three television viewing categories are presented in Tables 2 and 3 using an alpha level of 5% to determine statistical significance. Also, statistically significant differences in outcomes between categories of television viewing are indicated by differing superscripts, using an alpha level of 1% to partially adjust for multiple comparisons. All tests are 2-sided. Regression models also adjusting for Time 2 BMI did not differ from the models that did not adjust for BMI, therefore only data unadjusted for BMI are presented. Because the samples are racially and economically diverse, post-hoc analyses were completed to examine potential racial/ethnic and socioeconomic status interactions with Time 1 television use. Because of the post-hoc nature of these tests, a more conservative alpha level of 1% was used. All analyses were conducted using SAS version 9.1 (2003, SAS Institute, Cary, NC) and were weighted using the response propensity method [27] so the Project EAT-II sample resembled the Project EAT-I sample. With this method, the inverse of the estimated probability that an individual responded at Time 2 was used as the weight. Use of response propensity weights in this study helped influence estimates so that they are close to the estimates that would have been obtained had all of the students who participated at Time 1 completed surveys at Time 2. Concern regarding the missing-at-random assumption was lessened by the finding that after weighting and adjustment for demographic characteristics, baseline television viewing and dietary intake among responders were not significantly different from baseline television viewing and dietary intake among nonresponders of Project EAT-II.

**Table 2:** Dietary intake at Time 2 and television viewing behavior at Time 1 of younger cohort (middle school students at Time 1 and high school students at Time 2)^1^

	**Television Use^a,b^**						
		
	**Limited** **(<2 hrs/day)**		**Moderately high** **(2–5 hrs/day)**		**Heavy** **(≥5 hrs/day)**		
				
	**n**	**%**	**n**	**%**	**n**	**%**	
	176	31.2	270	47.9	118	20.9	
				
	**mean**	**SE**	**mean**	**SE**	**mean**	**SE**	***p *for linear trend**
	
Total energy (kcal/day)	2159.2	69.3	2025.3	55.4	2217.4	85.5	.98
Fruit (serv/day)	2.06^a^	.09	1.91^a,b^	.07	1.72^b^	.11	.009
Vegetable (serv/day)^2^	1.65	.08	1.65	.07	1.56	.10	.21
Whole grains (serv/day)^3^	.58	.06	.64	.04	.58	.07	.92
Calcium-rich foods (serv/day)	2.94	.09	2.97	.07	3.15	.11	.28
% total fat	30.5	.3	30.5	.3	29.8	.4	.25
% saturated fat	10.8	.2	10.8	.1	10.6	.2	.47
% trans fat	1.3	.02	1.3	.02	1.3	.03	.51
Fried food (serv/day)	.55	.02	.56	.02	.54	.03	.84
Fast food (times/week)	2.22	.08	2.29	.06	2.32	.10	.55
Snack food (serv/day)	2.33	.09	2.33	.07	2.53	.12	.74
Sugar-sweetened beverage (serv/day)	1.24^a,b^	.07	1.23^a^	.05	1.48^b^	.08	.02

^a,b ^Estimates in the same row marked with different letters are statistically significantly different from each other (p < .01).

^1 ^Multiple regression models are adjusted for race/ethnicity, gender, socioeconomic status, total energy intake at Time 2, and dietary intake at Time 1.

^2 ^Vegetable intake does not include servings of French fries.

^3 ^Whole grains intake does not include chip servings.

**Table 3:** Dietary intake at Time 2 and television viewing behavior at Time 1 of older cohort (high school students at Time 1 and young adults at Time 2)^1^

	**Television Use^a,b^**						
		
	**Limited** **(<2 hrs/day)**		**Moderately high** **(2–5 hrs/day)**		**Heavy** **(≥5 hrs/day)**		
				
	**n**	**%**	**n**	**%**	**n**	**%**	
	486	35.6	664	48.6	216	15.8	
				
	**mean**	**SE**	**mean**	**SE**	**mean**	**SE**	***p *for linear trend**
	
Total energy (kcal/day)	1837.1	33.7	1830.6	28.5	2041.0	50.2	.05
Fruit (serv/day)	1.70^a^	.05	1.60^a^	.04	1.25^b^	.07	<.001
Vegetable (serv/day)^2^	1.71^a^	.04	1.54^a^	.04	1.28^b^	.07	<.001
Whole grains (serv/day)^3^	.74^a^	.04	.64^b^	.03	.59^b^	.05	.002
Calcium-rich foods (serv/day)	2.78^a^	.05	2.67^a,b^	.04	2.50^b^	.07	<.001
% total fat	29.6	.2	30.0	.2	30.6	.3	.02
% saturated fat	10.3	.1	10.4	.09	10.5	.2	.23
% trans fat	1.2^a^	.01	1.2^a,b^	.01	1.3^b^	.02	.005
Fried food (serv/day)	.43^a^	.01	.47^b^	.01	.51^b^	.02	.001
Fast food (times/week)	2.03^a^	.04	2.17^b^	.03	2.33^b^	.06	<.001
Snack food (serv/day)	1.93^a^	.05	1.89^a^	.04	2.20^b^	.07	.012
Sugar-sweetened beverage (serv/day)	1.14^a^	.04	1.22^a,b^	.03	1.33^b^	.06	.004

^a,b ^Estimates in the same row marked with different letters are statistically significantly different from each other (p < .01).

^1 ^Multiple regression models are adjusted for race/ethnicity, gender, socioeconomic status, total energy intake at Time 2, and dietary intake at Time 1.

^2 ^Vegetable intake does not include servings of French fries.

^3 ^Whole grains intake does not include chip servings.

## Results

Among the younger cohort (middle school students at Time 1), 31.2% reported limited television use (<2 hours/day), 47.9% moderately high use (2–5 hours/day), and 20.9% heavy use (≥5 hours/day) at Time 1. Time 1 heavy television viewers reported lower fruit intake at Time 2 than Time 1 limited television viewers and higher sugar-sweetened beverage consumption than Time 1 moderately high television viewers. No other differences in dietary intake by television viewing categories were found among the younger cohort.

Among the older cohort (high school students at Time 1), 35.6% were limited, 48.6% were moderately high, and 15.8% were heavy television viewers at Time 1. Television viewing while in high school was associated with the consumption of several foods five years later (Table 3). Time 1 heavy television viewers reported lower intakes of fruits and vegetables, and higher consumption of snack foods, as compared to Time 1 limited and moderately high television viewers. Time 1 heavy television viewers reported fewer servings of calcium-rich foods, greater percentage of total calories from trans fat, and greater servings of sugar-sweetened beverages at Time 2, as compared to Time 1 limited television viewers. Additionally, participants who watched more than 2 hours of television while in high school reported consuming less servings of whole grains and more servings of fried foods, and ate more times per week at a fast food restaurant than participants who watched <2 hours of television at Time 1.

Post-hoc analyses revealed that race/ethnicity, but not socioeconomic status, interacted with the relationship between Time 1 television use and Time 2 dietary intake for participants in the older cohort. Black and Hispanic participants who watched more than five hours of television when they were in high school reported significantly greater total energy intake than white participants (2683 kcal and 2748 kcal, respectively, compared to 1908 kcal; p = .01). Black participants who watched fewer than 2 hours of television while in high school reported a higher calcium-rich food intake than white participants (2.9 servings compared to 2.2 servings; p = .006). Among participants in the younger cohort, neither race/ethnicity nor socioeconomic status interacted with the relationship between Time 1 television use and any of the dietary behavior outcomes.

## Discussion

The current study examined the longitudinal relationship between television viewing of adolescents in middle school and high school and their dietary intake five years later. Among participants in the older cohort, those who watched more than five hours of television per day when they were in high school reported less healthful eating habits (lower intakes of fruits, vegetables, whole grains and calcium-rich foods, and higher intakes of snack foods, fried foods, fast food, sugar-sweetened beverages, and trans fat). In contrast, television viewing in middle school only predicted lower fruit and greater sugar-sweetened beverage consumption five years later. Thus, television viewing during adolescence longitudinally predicted poorer dietary intake patterns five years later, with stronger and more consistent patterns seen during the transition from high school to young adulthood than during the transition from middle school to high school. Both time periods are critical developmental periods for adolescents, in which they are forming lifelong behaviors. However, behaviors exhibited in high school may more strongly influence behaviors reported in subsequent years than behaviors exhibited during middle school. Additionally, there may be environmental influences for which the current study did not have data, such as food and beverage television advertising, which may influence the eating behaviors of the cohorts differently.

Several cross-sectional studies have found a negative correlational association between television viewing and diet among students in preschool [28], elementary school [11,29,30], middle and high school [12,31,32], and college [33,34]. However, only two studies, both using data from Planet Health, which engaged middle school students in an obesity prevention intervention, have reported longitudinal relationships between greater amounts of television viewing and lower fruit and vegetable intake [13] and higher sweet and salty snack foods, fast food, and sugar-sweetened beverages [14] – results similar to those found in the current study. However, there are substantive differences between the Planet Health studies and the current study. Planet Health was an intervention that targeted reducing television viewing; the current study explored the influence of previous television viewing behavior over a longer period of time (5 years compared to 19 months); and the current study examined the transition from high school to the post-high school years, which to the authors' knowledge, has not been examined by any other research.

Among both cohorts, categories of food and beverages (snack foods, sugar-sweetened beverages, and fast foods) commonly advertised on television [7] were associated with previous television viewing behavior in the current study. Healthy foods, such as fruits, vegetables, whole grains, milk, and low-fat items are rarely advertised on television [6]. Repeated exposure to high calorie, low nutrient foods may increase desire for these foods and subsequently purchases and consumption of advertised products. Adolescents who watch too much television become adults who watch too much television [35], and thus continue to be exposed to advertisements for unhealthy foods. Although young people may be aware that many foods advertised on television are not healthy, they may chose to ignore or do not fully realize the consequences, because the actors they see advertising and eating the foods in the commercials are usually not overweight [36].

Post-hoc analyses in the older cohort showed there was an interaction with race/ethnicity in the observed relationship between television viewing and dietary intake. The stronger influence found in minority, especially black, participants may reflect the types of advertisements or program content most likely to reach these racial/ethnic groups. More food advertisements that focus on fast food, candy, and soda are shown during programs targeted to black Americans than programs targeted to general audiences [37].

There is also the issue of eating while viewing television. Studies have found that for some adolescents, a significant proportion of their total daily energy intake is consumed while viewing television [30,38]. Viewing television while eating may cause a distraction resulting in a lack of awareness of actual food consumption or overlooking satiety cues [33,39], which may lead to overconsumption. If these habits are formed at a younger age, they may continue as adolescents become older.

To our knowledge, this is the first study to examine the long-term longitudinal association between television viewing and dietary intake during the transition through the adolescent years into young adulthood. The strengths of this study are a prospective design; use of large, socioeconomically and racially diverse samples; and the examination of multiple dietary behaviors in two age cohorts. A limitation of the current study is that specific mechanisms through which television viewing may be influencing dietary behavior cannot be examined with these data. For example, the type and content of commercials watched by the participants are unknown. Future research is needed to explore these issues in more detail. Although the longitudinal nature of the current study allows for the assessment of temporality of the association between television viewing and dietary behavior, it does not allow for an assessment of causality. Television use may be overestimated in this study because the measure used to assess television use also included video use. It is unknown whether television viewing and video viewing influence dietary behaviors differently. However, among adolescents the proportion of total screen time spent watching videos is small compared to the proportion of time spent watching television [3].

## Conclusion

Findings from this study indicate that television viewing during adolescence, especially viewing during the high school years, predicts future eating habits. The research presented in this article begins to explore the television-diet paradigm of adolescents as they transition into young adulthood. However, from the current analyses, it cannot be determined if the relationship is causal and it cannot be determined the extent to which specific mechanism(s) are driving the association. Previous work in this area has proposed that the mechanisms through which television viewing influences obesity are: displacement of physical activity, promotion of increased energy intake while watching television, promotion of increased energy intake through advertising, and reduction in resting metabolic rate [40]. However, further examination has indicated that the association between television viewing and physical activity is weak [41]. Increasingly, researchers have begun to propose that television advertising and foods eaten in front of the television may be the primary underlying mechanism driving this relationship [28,30,42].

Adolescents are one of the primary targets of food and beverage advertising and television continues to be the main form of media for advertising to the general public [4]. There are potential negative impacts of advertising and marketing campaigns on dietary quality and food purchasing behavior during adolescence. Adolescence is a critical developmental period when individuals begin to make independent decisions about how to feed themselves. In particular, during the transition from high school to young adulthood youth begin to assume much greater responsibility for preparing or purchasing their own food, and potentially establish long-term adult eating behaviors. While much of the scientific discussion in this area, to date, has focused on short-term influences of television on dietary behavior, our work suggests that these associations are real and may, in fact, be sustained over the long-term. In addition to devising interventions to reduce television viewing time in adolescents, health professionals may need to develop interventions focusing on the promotion of healthy food choices, in general and while watching television, and overcoming media influences.

## Authors' contributions

DJBA conceived the current study, completed the data analysis, and drafted the manuscript. NIL assisted in the acquisition of the data and helped to draft and revise the manuscript. MCN helped to draft and revise the manuscript. DNS designed the larger studies (Project EAT I & II), acquired the data, and helped to revise the manuscript. MS assisted in the design of the larger studies (Project EAT I & II), assisted in the acquisition of the data, and helped to revise the manuscript.
